# Use of health and aged care services in Australia following hospital admission for myocardial infarction, stroke or heart failure

**DOI:** 10.1186/s12877-021-02519-w

**Published:** 2021-10-11

**Authors:** Benjumin Hsu, Rosemary J. Korda, Richard I. Lindley, Kirsty A. Douglas, Vasi Naganathan, Louisa R. Jorm

**Affiliations:** 1 Centre for Big Data Research in Health, UNSW Sydney, Sydney, NSW 2052 Australia; 2 School of Public Health, Faculty of Medicine and Health, University of Sydney, Sydney, Australia; 3 National Centre for Epidemiology and Population Health, Research School of Population Health, Australian National University, Canberra, Australia; 4 Westmead Applied Research Centre, Faculty of Medicine and Health, University of Sydney, Sydney, Australia; 5 The George Institute for Global Health, Sydney, New South Wales Australia; 6 ANU Medical School, College of Health and Medicine, Australian National University, Canberra, Australia; 7 Centre for Education and Research on Ageing, Concord Repatriation Hospital and University of Sydney, Sydney, Australia

**Keywords:** Cardiovascular, Care transitions, Community based long term care, Home care, Nursing home

## Abstract

**Background:**

Cardiovascular diseases (CVD), including myocardial infarction (MI), stroke and heart failure (HF) are the leading cause of death amongst the older population worldwide. The aim of this study is to investigate trajectories of use of health and aged care services after hospital admission for MI, stroke or HF among community-dwelling people not previously receiving aged care services.

**Methods:**

The study population comprised people aged 65+ years from the 45 and Up Study with linked records for hospital stays, aged care services and deaths for the period 2006–14. Among those with an index hospital admission for MI, stroke or HF, we developed Sankey plots to describe and visualize sequences and trajectories of service use (none, re-hospitalization, community care, residential care, death) in the 12 months following discharge. We used Cox proportional hazards models to estimate hazard ratios (HRs), for commencing community care and entering residential care (and the other outcomes) within 3, 6 and 12 months, compared to a matched group without MI, stroke or HF.

**Results:**

Two thousand six hundred thirty-nine, two thousand five hundred and two thousand eight hundred seventy-three people had an index hospitalization for MI, stroke and HF, respectively. Within 3 months of hospital discharge, 16, 32 and 29%, respectively, commenced community care (multivariable-adjusted HRs: 1.26 (95%CI:1.18–1.35), 1.53 (95%CI:1.44–1.64) and 1.39 (95%CI:1.32–1.48)); and 7, 18 and 14%, respectively, entered residential care (HRs: 1.25 (95%CI:1.12–1.41), 2.65 (95%CI:2.42–2.91) and 1.50 (95%CI:1.37–1.65)). Likewise, 26, 15 and 28%, respectively, were rehospitalized within 3 months following discharge (multivariable-adjusted HRs: 4.78 (95%CI:4.31–5.32), 3.26 (95%CI:2.91–3.65) and 4.94 (95%CI:4.47–5.46)).

**Conclusions:**

Older people hospitalized for major CVD may be vulnerable to transition-related risks and have poor health trajectories, thus emphasizing the value of preventing such events and care strategies targeted towards this at-risk group.

**Supplementary Information:**

The online version contains supplementary material available at 10.1186/s12877-021-02519-w.

## Background

Cardiovascular diseases (CVD), including myocardial infarction (MI), stroke and heart failure (HF) are the leading cause of death amongst the older population worldwide [1]. Despite declining CVD incidence and death rates in Australia, CVD is the second largest contributor to burden of disease [2]. Improvements in guideline-recommended interventions for these diseases have revolutionised management and improved clinical outcomes, including in-hospital mortality [3–5]. However, many older people who are hospitalized for CVD experience decline in functional independence, and hence require extra care and support after hospital discharge.

It is well known that stroke survivors in the older population are at an increased likelihood of being admitted directly to residential care at the time of hospital discharge [6]. Some studies have also suggested that there is an increased likelihood of residential care admission following MI or HF-related hospitalization [7–9]. Likewise, exacerbations and complications associated with MI, stroke and HF are common, and may result in rehospitalization [10–12]. However, there are critical knowledge gaps regarding health trajectories and transitions between health and aged care services following hospital admission for CVD in older people.

It is important to understand health trajectories and transitions following hospital discharge because it is expected to affect resource use and healthcare outcomes in older people. Through understanding the health trajectories over time, this allows anticipation of those at increased likelihood for using these services and may enhance better understanding of those that influence the expected transitions. In particular, health trajectories may inform health and social service planning to optimize quality of life and support people in their own homes. Hospitalization may precipitate discussion about moving into residential care, but older people prefer to remain living in the community because this enables them to maintain independence, autonomy and connection to friends and family [13]. Many older people fear being admitted to residential care, and admission may have significant psychological and social impacts, and reduce health-related quality of life [14]. Furthermore, people remaining in their homes and communities for as long as possible also minimises the societal economic costs associated with use of residential care [15, 16].

The primary aims of this study were to describe trajectories of use of aged care services and rehospitalization in the 12 months after index hospital admission for MI, stroke and HF among older community-dwelling people (65+ years), and to quantify the likelihood of commencing use of aged care services compared to those without MI, stroke and HF. Secondary aims were to quantify the likelihood of re-hospitalization and all-cause mortality following hospital admission for MI, stroke and HF, and to explore whether comorbidities or physical functioning might possibly account for any observed differences.

## Methods

### Study population

This study was part of the Exploring the Relationship between Social Care, Primary and Secondary Health Service Use and Adverse Health Outcomes (SHOut) project. The SHOut project uses baseline survey data from the Sax Institute’s 45 and Up Study linked to administrative data [17]. 45 and Up Study participants were from New South Wales (NSW), Australia, randomly sampled from the Department of Human Services (formerly Medicare Australia) enrolment database, which provides near complete coverage of the population. People 80+ years of age and residents of rural and remote areas were oversampled. A total of 266,942 participants (~ 18% of those invited, 11% of the NSW population aged 45 years and over) joined the study by completing a baseline questionnaire (between Jan 2006 and December 2009) and giving informed consent for follow-up and linkage of their information to routine health databases.

### Inclusion and exclusion

The final study population included people aged 65 years and over who did not self-report any heart diseases or stroke, and who had no hospitalization record with a diagnosis recorded in any field of MI, stroke or HF in the 2 years prior to completing the 45 and Up Study baseline survey (Supplementary Figure 1). Hence, participants between the age of 45 and 64 years, or had MI, stroke or HF during the look back period were excluded (*n* = 163,750). The 2 years look back period allows us to identify new onset of MI, stroke or HF more accurately. The study design timeline is provided in Supplementary Figure 2.

### Aged care

Data from the 45 and Up Study baseline survey were linked to the Pathways in Aged Care (PIAC) 2014 link map through the Australian Institute of Health and Welfare (AIHW) [18]. PIAC covers aged care assessments (required to access subsidized Australian care programs) and use of key aged care service programs, as well as deaths, to 30 June 2014 for all of Australia. The programs included are: Permanent Residential Aged Care; Respite Residential Aged Care; Community Aged Care Packages; Extended Aged Care At Home; Extended Aged Care At Home Dementia; Home Care Packages; Transition Care Program; and Home and Community Care.

Home and Community Care provides entry level services to clients in areas such as home maintenance, home modifications and aids to improve safety and access, meals, community transport, some nursing and allied health as well as day care services and respite care to assist carers. Home Care Packages aim to provide a more coordinated approach to support frail older people (65+ years or 50+ for Aboriginal or Torres Strait Islander peoples) living in the community. Transition Care Program provides home support services and rehabilitation for older people after a hospital stay. It provides short-term care for up to 12 weeks, including social work, nursing support, personal care and allied health care. Permanent Residential Aged Care refers to nursing homes (care homes) and people are eligible for this level of care if they need ongoing help with everyday tasks and/or health care. Respite Residential Aged Care provides formal care for a temporary period of time after which people usually return to living in the community. For this study, we categorized Community Aged Care Packages; Extended Aged Care At Home; Extended Aged Care At Home Dementia; Home Care Packages; Respite Residential Aged Care; Transition Care Program; and Home and Community Care as community care, and hereafter Permanent Residential Aged Care is referred to as residential care.

### Hospitalizations and all-cause mortality

The 45 and Up Study baseline survey was also linked to the NSW Admitted Patient Data Collection (APDC) and the Australian Coordinating Registry Cause of Death Unit Record File (COD-URF) from 01 July 2006 to 30 June 2014 through the Centre for Health Record Linkage (CHeReL). The APDC is a complete census of all public and private hospital admissions in NSW. Data include dates of admission and discharge, and the primary reason for admission using the International Classification of Diseases 10th revision – Australian, Modification (ICD-10-AM). This was used to identify patients with a coded diagnosis at discharge of myocardial infarction (MI, ICD-10: I21), stroke (ICD-10: I60-I64) or heart failure (HF; ICD-10: I50.0). The COD-URF provided data on fact and date of death.

### 45 and up study survey

The survey provided a range of sociodemographic data including age, sex (Male or Female), education (Did not complete high school (HS), HS/Apprenticeship/Diploma, or University of higher), household income (<$20,000, $20,000–$49,999, $50,000–$69,999, $70,000 or more, or Not specified), country of birth (Australia, or Other) and marital status (Single, Married/partner, or Widowed/divorced/separated).

Participants were also asked whether a doctor have had ever told them that they had any of the following conditions listed in the baseline survey questionnaire: heart disease, high blood pressure, stroke, diabetes, blood clot, asthma, Parkinson’s disease, and any cancer except skin cancer. A comorbidity score was calculated as the sum of all conditions reported.

Physical functioning was measured using the Medical Outcomes Study Physical Functioning (MOS-PF) scale, which indicates how participants’ health limits them in their daily functional activities [19]. Based on their score, participants were classified as having no functional limitation (≥81), minor/mild functional limitation (41–80), or moderate/severe functional limitation (≤40).

### Statistical analysis

We identified individuals who had an index hospitalization with a primary diagnosis of MI, stroke or HF who had not used aged care services in the 12 months prior to admission and who survived to hospital discharge. For each of these patient groups (MI, stroke or HF), we constructed a comparator group matched 3:1 on sex and age for the relevant index patient, sampled by optimal matching with replacement [20]. The descriptive characteristics of the study and comparator groups were summarized as means and standard deviations for continuous variables and as frequencies and percentage for categorical variables.

We described and visualised sequences and transitions of service use (none, re-hospitalization, community care, residential care, death) by 3-month intervals in the 12 months following discharge from hospital. We also stratified these analyses by sex and marital status. R version 3.6 and GoogleVis R package were used to develop Sankey plots for visualizing trajectory and transitions [21].

Hazard ratios (HRs) for commencing community-based aged care, entering residential aged care, re-hospitalization and dying within 3, 6 and 12 months for patients hospitalized due to MI, stroke or HF versus comparator group patients were estimated using Cox regression analysis. We conducted multivariable-adjusted analysis which included age, sex, education, household income, country of birth and marital status. We further explore whether comorbidities or physical functioning might possibly account for differences in use of aged care services between people with and without MI, stroke or HF by categorizing participants on basis of joint categories of MI, stroke or HF (yes/no) and comorbidity (None, 1, or 2+) and physical functioning (No limitation, Minor/Mild limitation, or Moderate/Severe limitation). These analyses were performed using SAS software 9.4 (SAS Institute Inc., Cary, NC, USA).

## Results

Baseline descriptive characteristics for patients hospitalized with MI, stroke or HF are shown in Table 1 and for the matched comparator groups in Supplementary Table 1. We identified 2639 participants with hospital diagnosis of MI, 2500 with stroke and 2873 with HF. The comparator group for MI included 7917 participants, 7500 for stroke and 8619 for HF. For both study and comparator groups, the mean age was 77 for MI, 78 for stroke and 80 for HF. Similarly for both groups, there were a greater proportion of male participants (65% in MI, 58% in stroke and 59% in HF. In general, participants in the comparator groups had better general health status and less comorbidities than the study groups.

**Table 1:** Baseline descriptive characteristics for patients admitted for myocardial infarction, stroke and heart failure

	Myocardial infarction (*n* = 2639)	Stroke (*n* = 2500)	Heart failure (*n* = 2873)
Age (years ± SD)	77.3 ± 7.1	78.4 ± 6.8	79.8 ± 6.8
Sex			
Male	1709 (65%)	1453 (58%)	1692 (59%)
Female	930 (35%)	1047 (42%)	1181 (41%)
Country of origin			
Australia	1885 (73%)	1768 (72%)	2070 (73%)
Other	711 (27%)	682 (28%)	751 (27%)
Education			
Did not complete high school (HS)	1149 (45%)	1081 (45%)	1382 (50%)
HS/Apprenticeship/Diploma	1089 (43%)	1011 (42%)	1058 (39%)
University of higher	296 (12%)	324 (13%)	301 (11%)
Income			
< $20,000	976 (42%)	932 (42%)	1168 (46%)
$20,000–$49,999	658 (28%)	613 (27%)	625 (25%)
$50,000–$69,999	100 (4%)	99 (4%)	106 (4%)
$70,000 or more	117 (5%)	127 (6%)	101 (4%)
Not specified	498 (21%)	466 (21%)	518 (21%)
Marital status			
Single	121 (5%)	117 (5%)	120 (4%)
Married/partner	1693 (64%)	1552 (62%)	1628 (57%)
Widowed/divorced/separated	813 (31%)	815 (33%)	1102 (39%)
Comorbidity			
None	1220 (46%)	1157 (46%)	1159 (40%)
1	974 (37%)	937 (37%)	1090 (38%)
2	379 (14%)	314 (13%)	479 (17%)
3 or more	66 (3%)	92 (4%)	145 (5%)
Body mass index			
Underweight	260 (10%)	303 (12%)	314 (11%)
Healthy weight	909 (35%)	937 (38%)	905 (32%)
Overweight	973 (37%)	880 (36%)	969 (34%)
Obese	463 (18%)	354 (14%)	641 (23%)
High blood pressure			
Yes	1407 (53%)	1257 (50%)	1600 (56%)
No	1232 (47%)	1243 (50%)	1273 (44%)
Diabetes			
Yes	516 (20%)	403 (16%)	659 (23%)
No	2123 (80%)	2097 (84%)	2214 (77%)
Self-reported health			
Excellent	124 (5%)	148 (6%)	68 (3%)
Very good	642 (26%)	600 (25%)	441 (17%)
Good	1033 (41%)	964 (41%)	1078 (41%)
Fair	561 (23%)	535 (23%)	843 (32%)
Poor	133 (5%)	109 (5%)	227 (9%)
Smoking status			
Never smoker	1416 (54%)	1404 (56%)	1497 (52%)
Past smoker	1079 (41%)	982 (39%)	1244 (44%)
Current smoker	131 (5%)	102 (4%)	106 (4%)
Anxiety and depression			
Low	1992 (81%)	1900 (82%)	2084 (78%)
Moderate	316 (13%)	281 (12%)	390 (15%)
High	101 (4%)	97 (4%)	134 (5%)
Very high	39 (2%)	32 (1%)	50 (2%)
Sufficient physical activity			
Yes	1250 (47%)	1223 (49%)	1134 (39%)
No	1389 (53%)	1277 (51%)	1739 (61%)
Safe alcohol drinking			
Yes	2096 (83%)	1966 (82%)	2246 (82%)
No	422 (17%)	436 (18%)	487 (18%)
Medication			
None	296 (11%)	287 (11%)	229 (8%)
1–4	1540 (58%)	1534 (61%)	1539 (54%)
5 or more	803 (30%)	679 (27%)	1105 (38%)

**Fig. 1:**
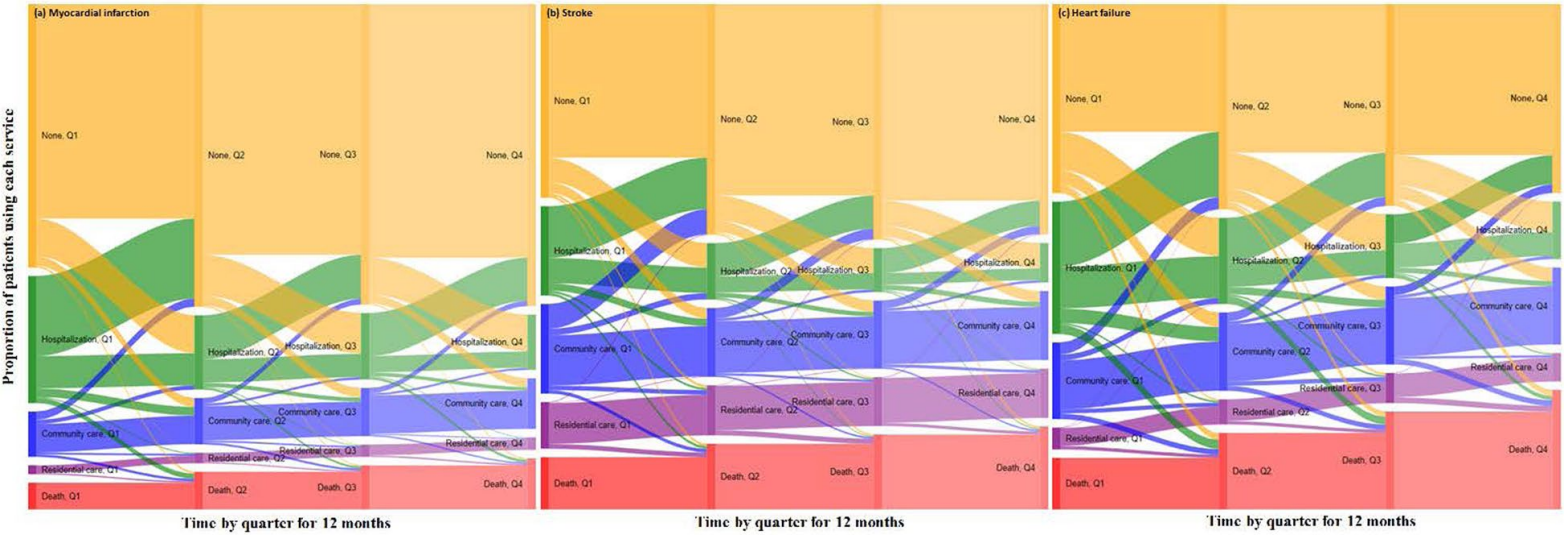
Transitions in service use (none, re-hospitalization, community aged care, residential aged care, death) by quarter for the 12 months post-discharge, for patients admitted with a primary diagnosis of myocardial infarction, stroke or heart failure

The distribution of service use over 12 months and the transitions between different services are shown in Fig. 1. Following an index admission for MI, stroke or HF, 16, 32 and 29% respectively, compared to 6, 14 and 10% of individuals, respectively, in the control groups commenced using community-based aged care within 3 months of hospital discharge and again for the majority of these people this service use was continued at 12 months post-discharge. Among MI, stroke and HF patients, 28, 47 and 52%, respectively, were using community-based aged care at 12 months post-discharge. Following an index admission for MI, stroke or HF, 7, 18 and 14% of individuals, respectively, entered residential aged care within 3 months of hospital discharge compared to 3, 5 and 4% in the control groups. The vast majority of these people remained there at 12 months post-discharge. Among MI, stroke and HF patients, 13, 28 and 30%, respectively, were using residential aged care at 12 months post-discharge. Larger proportions of individuals were re-hospitalized within 3 months of hospital discharge: 26, 15 and 28% for MI, stroke and HF, respectively. Over the 12 months post-discharge, 16, 29 and 34% of MI, stroke and HF patients died, while 46, 33 and 45% were re-hospitalized at least once. Of these, 23, 10 and 23% respectively were re-hospitalized at least once with a primary diagnosis of CVD.

Use of and transitions between services over time varied by sex and marital status (Supplementary Figures 3–4). Larger proportions of men than women were re-hospitalized, whereas a larger proportion of women either commenced community-based aged care or entered residential aged care following hospital discharge for MI or HF. Greater proportions of patients without a partner or spouse commenced using community aged care or residential care compared with patients who had a partner or spouse.

Patients hospitalized with MI, stroke and HF, compared to matched individuals who had not had a relevant index admission or used aged care services in the prior 12 months, had significantly elevated likelihood of using community-based aged care services within 3 months (respective multivariable-adjusted HRs: 1.26, 95%CI:1.18–1.35; 1.53, 95%CI:1.44–1.64; and 1.39, 95%CI:1.32–1.48) and entering residential aged care within 3 months (respective multivariable-adjusted HR: 1.25, 95%CI:1.12–1.41; 2.65, 95%CI:2.42–2.91; 1.50, 95%CI:1.37–1.65) (see Table 2 for community care and Table 3 for residential care). A significantly elevated likelihood of rehospitalization within 3 months (respective multivariable-adjusted HRs: 4.78, 95%CI: 4.31–5.32; 3.26, 95%CI: 2.91–3.65; 4.94, 95%CI: 4.47–5.46) and all-cause mortality within 3 months (respective multivariable-adjusted HRs: 1.59, 95%CI: 1.46–1.73; 1.88, 95%CI: 1.73–2.04; 2.31, 95%CI: 2.17–2.47) (see Table 4 for rehospitalization and Table 5 for mortality). Similar findings were observed for uptake of community-based, residential aged care services, rehospitalization and all-cause mortality at 6 and 12 months.

**Table 2:** Hazard ratios of using community aged care services 3 months, 6 months and 12 months after myocardial infarction, stroke and heart failure

	Unadjusted	Multivariable-adjusted^a^
Myocardial infarction		
3 months	1.29 (1.21–1.38)	1.26 (1.18–1.35)
6 months	1.36 (1.28–1.46)	1.34 (1.26–1.43)
12 months	1.42 (1.33–1.52)	1.40 (1.31–1.49)
Stroke		
3 months	1.54 (1.45–1.65)	1.53 (1.44–1.64)
6 months	1.68 (1.58–1.79)	1.67 (1.57–1.78)
12 months	1.76 (1.65–1.87)	1.75 (1.64–1.86)
Heart failure		
3 months	1.45 (1.36–1.53)	1.39 (1.32–1.48)
6 months	1.60 (1.51–1.69)	1.53 (1.45–1.63)
12 months	1.76 (1.66–1.87)	1.68 (1.59–1.78)

^a^Age, sex, education, household income, country of birth and marital status

**Table 3:** Hazard ratios of entering residential aged care 3 months, 6 months and 12 months after myocardial infarction, stroke and heart failure

	Unadjusted	Multivariable-adjusted^a^
Myocardial infarction		
3 months	1.30 (1.16–1.46)	1.25 (1.12–1.41)
6 months	1.34 (1.20–1.51)	1.30 (1.16–1.46)
12 months	1.39 (1.23–1.56)	1.35 (1.20–1.52)
Stroke		
3 months	2.65 (2.42–2.91)	2.65 (2.42–2.91)
6 months	2.80 (2.55–3.07)	2.83 (2.58–3.10)
12 months	2.91 (2.65–3.19)	2.94 (2.68–3.23)
Heart failure		
3 months	1.58 (1.45–1.74)	1.50 (1.37–1.65)
6 months	1.70 (1.55–1.86)	1.62 (1.47–1.77)
12 months	1.87 (1.70–2.04)	1.78 (1.62–1.95)

^a^Age, sex, education, household income, country of birth and marital status

**Table 4:** Hazard ratios of rehospitalization at 3 months, 6 months and 12 months after myocardial infarction, stroke and heart failure

	Unadjusted	Multivariable-adjusted^a^
Myocardial infarction		
3 months	4.70 (4.29–5.16)	4.78 (4.31–5.32)
6 months	3.54 (3.29–3.81)	3.54 (3.25–3.85)
12 months	2.55 (2.40–2.72)	2.56 (2.39–2.75)
Stroke		
3 months	3.14 (2.85–3.47)	3.26 (2.91–3.65)
6 months	2.41 (2.23–2.61)	2.47 (2.25–2.71)
12 months	1.82 (1.71–1.95)	1.82 (1.69–1.97)
Heart failure		
3 months	4.69 (4.31–5.11)	4.94 (4.47–5.46)
6 months	3.61 (3.37–3.86)	3.75 (3.46–4.06)
12 months	2.69 (2.54–2.85)	2.75 (2.57–2.94)

^a^Age, sex, education, household income, country of birth and marital status

**Table 5:** Hazard ratios of all-cause mortality at 3 months, 6 months and 12 months after myocardial infarction, stroke and heart failure

	Unadjusted	Multivariable-adjusted^a^
Myocardial infarction		
3 months	1.64 (1.51–1.78)	1.59 (1.46–1.73)
6 months	1.68 (1.54–1.82)	1.63 (1.50–1.77)
12 months	1.72 (1.59–1.87)	1.68 (1.55–1.83)
Stroke		
3 months	1.91 (1.76–2.07)	1.88 (1.73–2.04)
6 months	1.96 (1.81–2.13)	1.94 (1.79–2.10)
12 months	2.03 (1.87–2.20)	2.00 (1.85–2.17)
Heart failure		
3 months	2.37 (2.22–2.53)	2.31 (2.17–2.47)
6 months	2.50 (2.34–2.67)	2.44 (2.28–2.61)
12 months	2.71 (2.54–2.90)	2.65 (2.48–2.83)

^a^Age, sex, education, household income, country of birth and marital status

The likelihood of commencing community care or entering residential care increased with increasing levels of physical functioning limitations and, to a lesser extent, number of comorbidities, among participants with and without MI, stroke or HF (Supplementary Tables 2 and 3). Thus, HRs were particularly high for those with MI, stroke and HF who had poor physical health (respective multivariable-adjusted HRs: 3.37, 95%CI: 2.89–3.93; 3.83, 95%CI: 3.30–4.45; 3.28, 95%CI: 2.87–3.75). Among participants with no functional limitations and no comorbidities, MI, stroke of HF hospitalization remained associated with elevated likelihood of commencing aged care services.

## Discussion

Our study for the first time quantified and visualised trajectories of initiation and use of community-based and residential aged care services following hospitalization for major CVD – MI, stroke and HF. We identified substantial elevated likelihood of initiation of community-based and residential aged care across all three major CVD conditions. Further we found that almost all individuals who commenced using aged care services after hospitalization for these major CVD conditions continued to do so at 12 months post-discharge. In particular, patients hospitalized for stroke or HF had a high probability of using community-based (47 and 52%, respectively) and residential aged care (28 and 30%, respectively) at 12 months post-discharge. Furthermore, patients hospitalized for these major CVD conditions had a high probability of being re-hospitalized (33% in stroke, 45% in HF and 46% in MI) at least once.

It is well established that residential care admission after stroke is common, particularly in those with older age and greater stroke severity [6]. However, the existing scientific literature on the likelihood of entering residential care following MI and HF varies. Similar to our findings, a study using Danish nationwide registries (*n* = 26,539) showed older patients (65+ years) discharged following MI hospitalization compared with all other hospitalization were at an increased likelihood of subsequent residential care admission [7]. Likewise, a Finnish study using linked health administrative datasets (*n* = 301,263) also showed older patients (65+ years) were at an increased likelihood of entering residential care following hospitalization due to heart disease, including both MI and HF [8]. However, a German study using health insurance data (*n* = 414,049) showed older patients (66+ year) had a lower likelihood of entering residential care following MI hospitalization compared with all other hospitalization other than femoral fracture, stroke and pneumonia [22]. Whereas a Dutch study using data from three national databases (*n* = 262,439) showed older patients (65+ years) with a hospital admission for HF compared with all other hospitalization may be at an increased likelihood of entering low-level residential care but a lower likelihood of entering high-level residential care [9]. These varied findings may reflect differences in aged care service provision between settings, as well as in cohort characteristics, reference groups and follow-up. In particular, our reference group came from a community-dwelling population-based cohort, rather than being drawn from people admitted to hospital for other conditions.

The current literature also shows that patients hospitalized with MI, stroke and HF have increased use of formal home and community care services over time following discharge [23–26]. In addition, transitional care services such as skilled nursing facilities in the United States have also been reported to be commonly used by patients following cardiac hospitalization [27, 28]. The Dutch study using data from three national databases showed older patients discharged with a diagnosis of HF or cerebrovascular disease were at an increased risk of commencing home care services compared with patients discharged without a diagnosis of these conditions [9].

Our findings suggest that older people with CVD compounded by functional limitations and comorbidities may be particularly vulnerable as they have a greater likelihood of commencing the use of aged care services. Furthermore, it is known from the existing literature, and confirmed in this study, that rehospitalization rates are high in older adults following hospital discharge due to major CVD conditions [29–31]. Hence, the discharge from hospital back to community is a crucial period for older adults to minimize the risk of rehospitalization and adverse health outcomes, particularly for those with frailty and multimorbidity. A prospective multicentre study of 906 HF patients demonstrated that assessment of patients within 7 days by cardiologists, general practitioners or heart failure nurses post-discharge significantly reduced rates of rehospitalization [32]. Thus, coordination of primary care, allied health and other support services can help to ensure care when older cardiovascular patients transfer across care settings and support reintegration back into everyday activities and improved quality of life [33]. Successful care models are those that comprehensively target all of the risk factors that impact on cardiovascular health with clear action plans, goal-setting approaches and proven therapeutics [34, 35]. Some Australian Local Health Districts and Primary Health Networks currently provide outreach services to residential aged care for chronic disease management, such as Geriatric Flying Squad and remote physiological monitoring coupled with telehealth [36–38].

Primary care is well placed for the care of older people with its strengths of having a multidisciplinary management focus on secondary prevention, and knowledge of the patient including their social context and capacity for ongoing chronic disease management [39]. However, patients may lose connection with their primary care provider at points of transition such as entry to residential aged care [40]. A population-based cohort study in Canada (*n* = 50,089) has shown that only 12% of residents retained their family physicians after entry into residential care facilities [41]. Likewise, a recent report using routinely collected linked health administrative data for more than 37,300 coronary heart disease patients showed timely and regular contact with a general practitioner were associated with lower risk of having an emergency rehospitalization due to CVD [42]. The reduced relational contact with primary care provider may worsen health issues during this transition.

It is important for older adults in aged care to be provided with evidence-based recommended care to improve their health outcomes. However, previous studies have shown that people living in the general community are more likely to receive stroke rehabilitation processes of care than people using residential care [43], and older adults in residential care often do not receive MI or HF guideline-recommended management [44–47]. Importantly, current clinical guidelines and recommendations for CVD regarding pharmacological and non-pharmacological treatment after hospital discharge do not account for age and geriatric syndromes [48, 49]. It may be difficult for health professionals to provide the most appropriate management to older adults based on a single disease guideline due to the conflicting established evidence on the clinical consequences of polypharmacy and multimorbidity in older adults [50, 51]. The lower adherence to clinical guidelines in people using aged care may be due to a paucity of data to guide optimal management for this specific population. In general, older adults using aged care services are rarely included in clinical trials, subsequently causing problems of generalisability of evidence from the secondary CVD prevention literature [52]. There are significant knowledge gaps in understanding how these mainstay rehabilitation and secondary prevention services affect outcomes in the aged care population. Further studies are warranted to explore whether the existing rehabilitation and prevention services for older adults focus older patients in improving quality of life and regaining functional abilities to ensure optimal recovery leading to independence and autonomy [53].

Finally, our findings show that greater proportions of older patients, women, those living without a partner or spouse commence using community aged care or enter residential care after hospital discharge from MI, stroke, and HF. This may reflect the lower levels of carer support at home. Post-discharge cardiovascular support for older female patients and patients of both sexes living without a partner may present a significant opportunity for reducing premature transitions into community aged care or residential care. Nevertheless, future studies are warranted to disentangle the effect between older age, sex and marital status with the increased likelihood of using health and aged care services.

The major strength of this study includes its large sample size, permitting generation of a comprehensive profile of the use of community aged care and residential care after hospital discharge from MI, stroke or HF in the Australian context. Another strength was the use of linked routinely- collected administrative health data which allowed ascertainment of cardiovascular hospitalizations, along with the use of government-funded aged care services. However, our linked data did not capture use of self-funded aged care services and informal care. As a result, periods of no service use may have in fact represented periods in which self-funded aged care services or informal care were being used, potentially attenuating differences between the cardiovascular patients and the comparator groups. Furthermore, number of people using community and residential care in Australia have continued to increase and the pattern of care usage has changed since our study period (2006–14), suggesting that the burden of using health and aged care services now may be slightly different [54]. More up to date linked health and aged care data are warranted to confirm our observations and findings in order to improve health policy and drive clinical practice change. Nevertheless, our study provides a comprehensive novel overview of the use of health and aged care services following hospital admission for major cardiovascular diseases in older Australian people. Some members of our study population may have had hospitalizations for MI, stroke or HF prior to the 2-year exclusion period. As a result, some index hospitalizations may not have been first hospitalizations, and some comparator group members may have had prior hospitalizations, potentially attenuating differences between groups. Finally, measurement bias is inevitable in our self-reported comorbidities and physical functioning status.

## Conclusion

Older people hospitalized for major CVD may be vulnerable to transition-related risks, substantially elevated likelihood of commencing aged care services, and poor health trajectories after discharge from hospital, thus emphasizing the value of preventing such events and care strategies targeted towards this at-risk group.

## Supplementary Information

**Additional file 1 **: **Table S1**. Baseline descriptive characteristics for the matched comparator groups for patients admitted with myocardial infarction, stroke and heart failure. **Table S2**. Multivariable-adjusted hazard ratios for using community care at 12 months after myocardial infarction, stroke or heart failure according to joint categories of physical functioning limitations and comorbidities and the respective cardiovascular conditions. **Table S3**. Multivariable-adjusted hazard ratios for entering residential care at 12 months after myocardial infarction, stroke or heart failure according to joint categories of physical functioning limitations and comorbidities and the respective cardiovascular conditions.

**Additional file 2 **: **Figure S1**. Study flow chart. **Figure S2**. Study design timeline. **Figure S3**. Transitions in service use (none, re-hospitalization, community aged care, residential aged care, death) by quarter for the 12 months post-discharge, for patients admitted with a primary diagnosis of myocardial infarction, stroke or heart failure, by sex. **Figure S4**. Transitions in service use (none, re-hospitalization, community aged care, residential aged care, death) by quarter for the 12 months post-discharge, for patients admitted with a primary diagnosis of myocardial infarction, stroke or heart failure, by marital status.

## Data Availability

The data that support the findings of this study are available from the Sax Institute but restrictions apply to the availability of these data, which were used under license for the current study, and so are not publicly available. Data are however available from the authors upon reasonable request and with permission of the Sax Institute.
