# Supplementary material for: Use of health and aged care services in Australia following hospital admission for myocardial infarction, stroke or heart failure
Source: BMC Geriatr. 2021 Oct 11;21:538. doi: 10.1186/s12877-021-02519-w (PMC8504055; doi:10.1186/s12877-021-02519-w)
Supplement: Supplementary file 1 — Additional file 1 : Table S1. Baseline descriptive characteristics for the matched comparator groups for patients admitted with myocardial infarction, stroke and heart failure. Table S2. Multivariable-adjusted hazard ratios for using community care at 12 months after myocardial infarction, stroke or heart failure according to joint categories of physical functioning limitations and comorbidities and the respective cardiovascular conditions. Table S3. Multivariable-adjusted hazard ratios for entering residential care at 12 months after myocardial infarction, stroke or heart failure according to joint categories of physical functioning limitations and comorbidities and the respective cardiovascular conditions. [file 12877_2021_2519_MOESM1_ESM.docx]

SUPPLEMENTARY TABLE 1. Baseline descriptive characteristics for the matched comparator groups for patients admitted with myocardial infarction, stroke and congestive heart failure

|  | Myocardial infarction  (n=7917) | Stroke  (n=7500) | Heart failure  (n=8619) |
| --- | --- | --- | --- |
| Age (years ± SD) | 77.3 ± 7.1 | 78.4 ± 6.8 | 79.8 ± 6.8 |
| Sex  Male  Female | 5127 (65%)  2790 (35%) | 4359 (58%)  3141 (42%) | 5076 (59%)  3543 (41%) |
| Country of origin  Australia  Other | 5630 (72%)  2180 (28%) | 5374 (73%)  2015 (27%) | 6123 (72%)  2375 (28%) |
| Education  Did not complete high school (HS)  HS/Apprenticeship/Diploma  University of higher | 3185 (42%)  3244 (42%)  1238 (16%) | 3202 (44%)  3010 (41%)  1055 (15%) | 3637 (44%)  3409 (41%)  1278 (15%) |
| Income  <$20000  $20000-$49999  $50000-$69999  $70000 or more  Not specified | 2643 (36%)  2228 (31%)  438 (6%)  496 (7%)  1460 (20%) | 2581 (38%)  2010 (30%)  326 (5%)  470 (7%)  1396 (21%) | 3019 (39%)  2211 (29%)  424 (5%)  511 (7%)  1576 (20%) |
| Marital status  Single  Married/partner  Widowed/divorced/separated | 335 (4%)  5239 (67%)  2285 (29%) | 360 (5%)  4727 (63%)  2364 (32%) | 406 (5%)  5276 (62%)  2873 (34%) |
| Comorbidity  None  1  2  3 or more | 4140 (52%)  2855 (36%)  778 (10%)  144 (2%) | 4003 (53%)  2620 (35%)  730 (10%)  147 (2%) | 4507 (52%)  3064 (36%)  867 (10%)  181 (2%) |
| Body mass index  Underweight  Healthy weight  Overweight  Obese | 755 (10%)  2917 (37%)  3012 (38%)  1152 (15%) | 783 (11%)  2872 (39%)  2692 (36%)  1078 (15%) | 949 (11%)  3572 (42%)  2940 (34%)  1074 (13%) |
| High blood pressure  Yes  No | 3715 (47%)  4202 (53%) | 3629 (48%)  3871 (52%) | 4032 (47%)  4587 (53%) |
| Diabetes  Yes  No | 1027 (13%)  6890 (87%) | 950 (13%)  6550 (87%) | 1050 (12%)  7569 (88%) |
| Self-reported health  Excellent  Very good  Good  Fair  Poor | 676 (9%)  2452 (33%)  2918 (39%)  1252 (17%)  202 (3%) | 599 (8%)  2218 (31%)  2859 (40%)  1215 (17%)  210 (3%) | 706 (9%)  2495 (31%)  3235 (40%)  1476 (18%)  235 (3%) |
| Smoking status  Never smoker  Past smoker  Current smoker | 4398 (56%)  3222 (41%)  251 (3%) | 4326 (58%)  2873 (39%)  245 (3%) | 4990 (58%)  3326 (39%)  252 (3%) |
| Anxiety and depression  Low  Moderate  High  Very high | 6332 (84%)  845 (11%)  236 (3%)  86 (1%) | 6004 (85%)  752 (11%)  218 (3%)  84 (1%) | 6868 (8%%)  870 (11%)  272 (3%)  85 (1%) |
| Sufficient physical activity  Yes  No | 4380 (55%)  3537 (45%) | 3956 (53%)  3544 (47%) | 4372 (51%)  4247 (49%) |
| Safe alcohol drinking  Yes  No | 6152 (80%)  1520 (20%) | 5913 (82%)  1323 (18%) | 6834 (82%)  1451 (18%) |
| Medication  None  1-4  5 or more | 1022 (13%)  5104 (64%)  1791 (23%) | 950 (13%)  4706 (63%)  1844 (25%) | 1107 (13%)  5474 (64%)  2038 (24%) |

SUPPLEMENTARY TABLE 2. Multivariable-adjusted* hazard ratios for using community care at 12 months after myocardial infarction, stroke or heart failure according to joint categories of physical functioning limitations and comorbidities and the respective cardiovascular conditions^†^

| **Community care** | Myocardial infarction | Stroke | Heart failure |
| --- | --- | --- | --- |
| **Physical function** |  |  |  |
| No condition and  No limitation  Minor/Mild limitation  Moderate/Severe limitation | 1.00  1.40 (1.20-1.63)  2.56 (2.22-2.95) | 1.00  1.27 (1.09-1.47)  2.34 (2.04-2.68) | 1.00  1.25 (1.09-1.43)  2.18 (1.92-2.48) |
| Yes condition and  No limitation  Minor/Mild limitation  Moderate/Severe limitation | 1.49 (1.13-1.96)  2.01 (1.66-2.43)  3.37 (2.89-3.93) | 2.11 (1.65-2.70)  2.33 (1.93-2.81)  3.83 (3.30-4.45) | 2.36 (1.75-3.19)  2.52 (2.10-3.03)  3.28 (2.87-3.75) |
| **Comorbidity** |  |  |  |
| No condition and  No limitation  Minor/Mild limitation  Moderate/Severe limitation | 1.00  1.21 (1.11-1.33)  1.50 (1.37-1.65) | 1.00  1.14 (1.04-1.25)  1.38 (1.27-1.51) | 1.00  1.07 (0.99-1.16)  1.26 (1.16-1.36) |
| Yes condition and  No limitation  Minor/Mild limitation  Moderate/Severe limitation | 1.37 (1.17-1.61)  1.57 (1.38-1.79)  2.07 (1.86-2.30) | 1.68 (1.45-1.95)  2.07 (1.83-2.33)  2.29 (2.06-2.55) | 1.67 (1.42-1.96)  1.90 (1.77-2.12)  1.93 (1.77-2.12) |

*age, sex, education, household income, country of birth and marital status

^†^No condition indicates the participants do not have the respective cardiovascular condition (myocardial infarction, stroke or heart failure). Likewise, yes condition indicates the participants have the respective cardiovascular condition.

SUPPLEMENTARY TABLE 3. Multivariable-adjusted* hazard ratios for entering residential care at 12 months after myocardial infarction, stroke or heart failure according to joint categories of physical functioning limitations and comorbidities and the respective cardiovascular conditions^†^

| **Residential care** | Myocardial infarction | Stroke | Heart failure |
| --- | --- | --- | --- |
| **Physical function** |  |  |  |
| No condition and  No limitation  Minor/Mild limitation  Moderate/Severe limitation | 1.00  2.03 (1.38-3.00)  4.74 (3.30-6.81) | 1.00  1.00 (0.74-1.36)  2.57 (1.97-3.35) | 1.00  1.13 (0.87-1.48)  2.47 (1.95-3.12) |
| Yes condition and  No limitation  Minor/Mild limitation  Moderate/Severe limitation | 2.72 (1.54-4.82)  3.55 (2.30-5.47)  6.07 (4.17-8.82) | 3.34 (2.25-4.94)  4.81 (3.53-6.55)  6.46 (4.92-8.48) | 3.05 (1.84-5.05)  2.63 (1.89-3.65)  3.64 (2.85-4.65) |
| **Comorbidity** |  |  |  |
| No condition and  No limitation  Minor/Mild limitation  Moderate/Severe limitation | 1.00  1.05 (0.89-1.24)  1.21 (1.03-1.42) | 1.00  1.01 (0.86-1.18)  1.16 (1.00-1.36) | 1.00  0.94 (0.83-1.08)  1.05 (0.92-1.19) |
| Yes condition and  No limitation  Minor/Mild limitation  Moderate/Severe limitation | 1.63 (1.28-2.09)  1.47 (1.18-1.83)  1.43 (1.18-1.74) | 2.99 (2.44-3.65)  2.99 (2.51-3.56)  3.30 (2.81-3.86) | 2.11 (1.70-2.63)  1.82 (1.53-2.17)  1.68 (1.46-1.94) |

*age, sex, education, household income, country of birth and marital status

^†^No condition indicates the participants do not have the respective cardiovascular condition (myocardial infarction, stroke or heart failure). Likewise, yes condition indicates the participants have the respective cardiovascular condition.
