# Supplementary material for: Use of health and aged care services in Australia following hospital admission for myocardial infarction, stroke or heart failure
Source: BMC Geriatr. 2021 Oct 11;21:538. doi: 10.1186/s12877-021-02519-w (PMC8504055; doi:10.1186/s12877-021-02519-w)

SUPPLEMENTARY FIGURE 1. Study flow chart


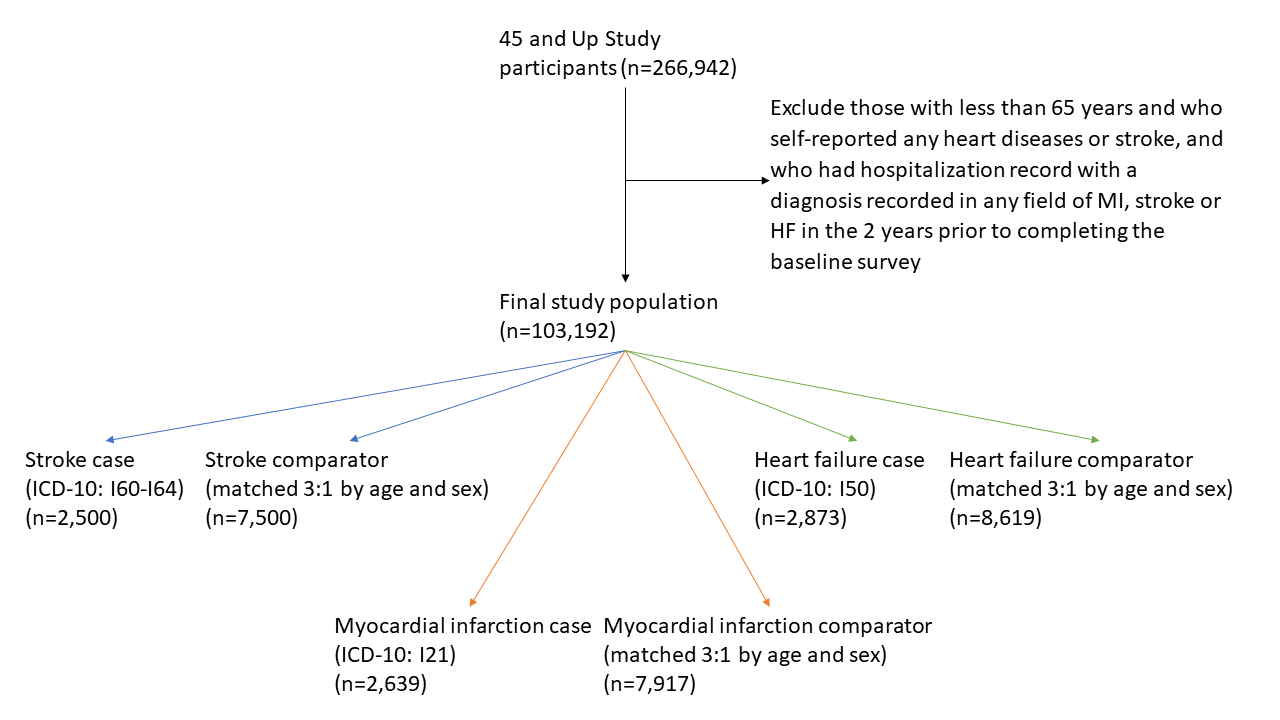


SUPPLEMENTARY FIGURE 2. Study desgin timeline


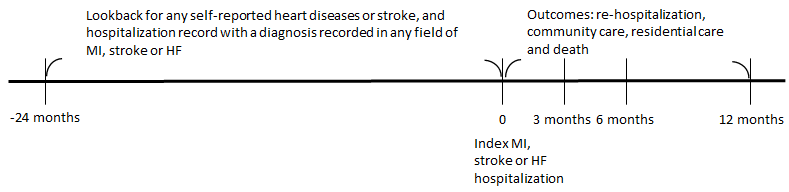


SUPPLEMENTARY FIGURE 3. Transitions in service use (none, re-hospitalization, community aged care, residential aged care, death) by quarter for the 12 months post-discharge, for patients admitted with a primary diagnosis of myocardial infarction, stroke or heart failure, by sex


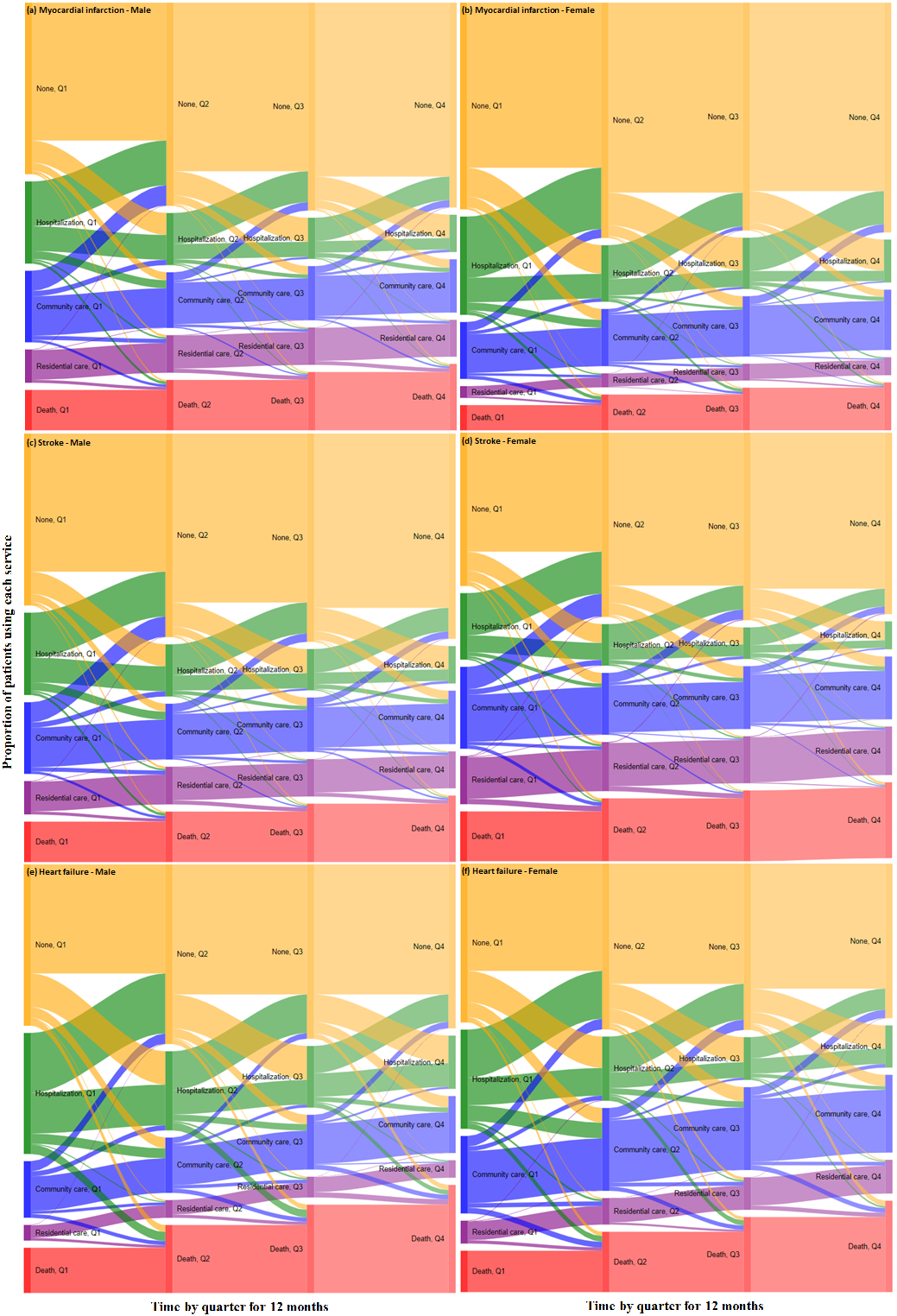


SUPPLEMENTARY FIGURE 4. Transitions in service use (none, re-hospitalization, community aged care, residential aged care, death) by quarter for the 12 months post-discharge, for patients admitted with a primary diagnosis of myocardial infarction, stroke or heart failure, by marital status


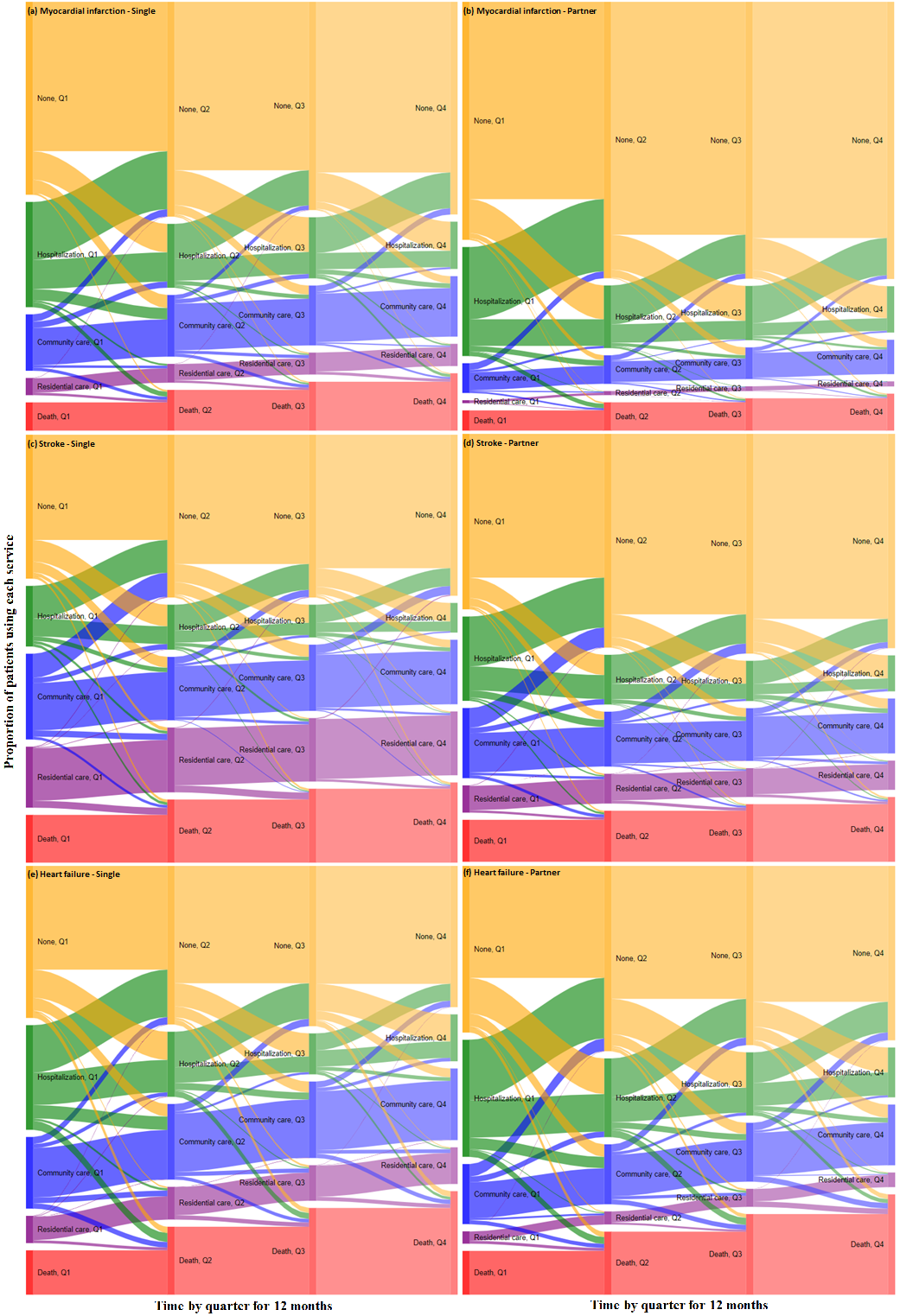

Supplement: Supplementary file 2 — Additional file 2 : Figure S1. Study flow chart. Figure S2. Study design timeline. Figure S3. Transitions in service use (none, re-hospitalization, community aged care, residential aged care, death) by quarter for the 12 months post-discharge, for patients admitted with a primary diagnosis of myocardial infarction, stroke or heart failure, by sex. Figure S4. Transitions in service use (none, re-hospitalization, community aged care, residential aged care, death) by quarter for the 12 months post-discharge, for patients admitted with a primary diagnosis of myocardial infarction, stroke or heart failure, by marital status. [file 12877_2021_2519_MOESM2_ESM.docx]
